# Medical Device Regulation – Who Does the FDA Serve?

**DOI:** 10.1017/jme.2025.10217

**Published:** 2026

**Authors:** Osman Moneer, Vinay Rathi

**Affiliations:** 1 Department of Medicine, Stanford University, United States; 2 Department of Otolaryngology – Head & Neck Surgery, The Ohio State University College of Medicine, United States

**Keywords:** Medical Device Regulation, FDA, 510(k)

## Abstract

Political pressures and institutional constraints have shaped a reactionary regulatory system that unduly preferences industry interests over public health. Breaking the reactive cycles that have long shaped device regulation will involve more than standard technical or incremental reforms. Congress would instead need to revisit more foundational values of device regulation to better align policy with the interests of patients and public health.

The US Food and Drug Administration (FDA) is charged with promoting patient access to novel devices and assuring these technologies are safe and effective. The article “Regulating Medical Devices: The Values and Politics of the US FDA Review Process” presents a sociohistorical narrative detailing the genesis of medical device regulation and FDA efforts to balance these often competing demands. Drawing on archival materials, oral histories from former FDA officials, Congressional records, and historical case studies, the authors argue that political pressures and institutional constraints have shaped a reactionary regulatory system that unduly preferences industry interests over public health.

The authors make convincing points. The FDA initially lacked formal authority over most devices and predominantly focused attention on fraudulent or harmful products already on the market. Congress only promulgated a regulatory framework in 1976 under mounting public pressure in the wake of high-profile device failures. By this time, complex medical devices were already deeply integrated into clinical practice, and the ensuing statute (the Medical Device Amendments of 1976) reflected political sensitivities to disrupting industry growth or slowing innovation. Facing considerable “regulatory debt” and capacity constraints, Congress and the FDA leaned heavily on existing industry standards and professional norms. These exigencies paved the way for the 510(k) process — which only requires that manufacturers demonstrate “substantial equivalence” to a previously cleared device for authorization — to unintentionally become the dominant route to market. Deference to industry status quo and burden were thus embedded statutory standards for devices from the outset.

The article omits two subsequent statutes that underscore the authors’ assertions about excessive political sensitivity to manufacturer regulatory burden and enthusiasm for new products. First, under the FDA Modernization Act of 1997, Congress directed FDA to require only the “least burdensome” information necessary during premarket evaluation to satisfy authorization standards.^1^ This legislation effectively prioritizes regulatory efficiency over scientific certainty. Second, under The Medical Device User Fee Amendments of 2002, Congress first authorized FDA to collect fees from manufacturers in exchange for application review time commitments. Successive legislative renewals have compressed FDA response and review times and rendered the agency increasingly reliant on industry fees.^2^ Laws obliging FDA to reduce manufacturer burden and depend on industry financing raise valid concerns about regulatory capture.

Though the authors provide a compelling historical synthesis, some of their concerns are overstated. For instance, FDA has taken steps to strengthen the 510(k) process (e.g., eliminating use of split or chimeric predicates) and is actively considering additional reforms, such as increasing premarket clinical data requirements and restricting the use of recalled predicates.^3^ Furthermore, the De Novo pathway is actually *more* rigorous than the 510(k) process; recent work demonstrates that over 80% of De Novo devices are supported by premarket clinical data (vs. ~10% for 510(k) devices) and none were subject to Class I (highest-risk) FDA recall.^4^ Finally, FDA downgrade of risk classification may be wholly appropriate for some devices based on accumulated regulatory and real-world experience.^5^

These subtleties are overshadowed by regulatory challenges not discussed within the article. As an example, “design drift” that compromises device safety and effectiveness is not unique to the 510(k) process. Under the PMA pathway, manufacturers can modify approved devices by submitting supplemental applications that rarely require clinical data to change technology design, manufacturing, or labeling. Permissive approval standards for high-risk device modifications can result in significant patient harm, such as failure to defibrillate or death in patients who were implanted with cardioverter–defibrillators that malfunctioned due to lead fracture and insulation failure.^6^ FDA often faces difficulty detecting and remedying such issues in the post-market setting due to systematic underreporting of adverse events,^7^ post-market study delays,^8^ and an inability to identify affected parties (e.g., patients and facilities) and communicate corrective actions.^9^ Moreover, most recalls are voluntarily initiated by manufacturers, to whom FDA largely delegates development and implementation of corrective action plans. Perhaps unsurprisingly, high-severity recalls may require several years to resolve, with one study showing a median of two years between recall initiation and termination.^10^

One limitation highlighted in the article remains a critical priority today: inadequate FDA workforce and resources.^11^ Qualified staff shortages, shortened response and review time goals, and increasing technological sophistication of devices have all strained the agency’s capacity. These constraints limit the FDA’s ability to conduct thorough reviews, enforce post-market commitments, and keep pace with emerging technologies, including software and AI-enabled devices. Notably, strengthening FDA staffing is one of the few priorities on which industry, public health advocates, and the agency are all aligned. Without sufficient human capital, the potential for even well-designed reforms to advance the agency’s mission would be blunted.

The article provides a compelling sociohistorical analysis illuminating how and why FDA has catered to the device industry over the past 50 years. The authors’ central insight on how political values, institutional pressures, and historical contingencies can render regulation that prioritizes access over evidence resonates strongly with contemporary affairs. Breaking the reactive cycles that have long shaped device regulation will involve more than standard technical or incremental reforms. Congress would instead need to revisit more foundational values of device regulation to better align policy with the interests of patients and public health.
